# Quick evaluation of lower leg ischemia in patients with peripheral arterial disease by time maximum intensity projection CT angiography: a pilot study

**DOI:** 10.1186/s12880-020-00537-5

**Published:** 2021-01-06

**Authors:** Daming Zhang, Xueyan Zhou, Haiping Zhang, Xiaobing Fan, Zehong Lin, Huadan Xue, Yining Wang, Zhengyu Jin, Yuexin Chen

**Affiliations:** 1 Department of Radiology, Peking Union Medical College Hospital, Chinese Academy of Medical Sciences and Peking Union Medical College, Beijing, China; 2 School of Technology, Harbin University, Harbin, China; 3 Department of Radiology, Beijing Friendship Hospital, Capital Medical University, Beijing, China; 4 Department of Radiology, The University of Chicago, Chicago, IL 60637 USA; 5 Department of Vascular Surgery, Peking Union Medical College Hospital, Chinese Academy of Medical Sciences and Peking Union Medical College, Beijing, China

**Keywords:** Peripheral arterial disease, Computed tomographic angiography, Maximum intensity projection

## Abstract

**Background:**

The purpose of this study is to evaluate a new method involving time maximum intensity projection (t-MIP) postprocessed from dynamic computed tomographic angiography (dyn-CTA) in diagnosing peripheral arterial disease (PAD).

**Methods:**

A population of 34 patients with known PAD was examined with a combined CTA protocol consisting of a standard CTA (s-CTA) scan of the lower extremities and a dyn-CTA scan of the calves. For each lower leg, t-MIP images consisting of the MIP_0_ (sagittal MIP), MIP_+θ_ (45° lateral MIP), and MIP_−θ_ (− 45° lateral MIP) were automatically generated from dyn-CTA. An objective evaluation of the vascular CT attenuation of the best enhancement phase of dyn-CTA and t-MIP was measured; a subjective evaluation of vessel stenosis and occlusion was performed, assigning a score for t-MIP and s-CTA. The CT attenuation of t-MIP and dyn-CTA was compared, as were the runoff scores of t-MIP and s-CTA.

**Results:**

The CT attenuation of t-MIP CTA of three vascular segments from 68 lower extremities was higher than that of the best enhancement phase of dyn-CTA and s-CTA, with statistically significant differences at the posterior tibial artery and fibular artery (all *p* < 0.05). There were strong correlations (r ≥ 0.75, p < 0.05) of the runoff scores between t-MIP and s-CTA.

**Conclusions:**

There is potential clinical applicability of t-MIP in assisting with the diagnosis of lower leg vascular stenosis in dyn-CTA with reliable diagnostic accuracy and convenient immediacy.

## Background

Peripheral arterial disease (PAD) of the lower extremities is frequently underdiagnosed [1], partly due to the wide variety of lower extremity symptoms that PAD patients exhibit and partly due to the high prevalence of asymptomatic PAD [2].

Currently, runoff computed tomographic angiography (CTA) of the peripheral vessels has become a widely used diagnostic option for patients with PAD. Runoff CTA, which is also called standard CTA (s-CTA), is often incorporated into patients’ treatment planning because it is accessible, quick and relatively inexpensive. CTA can provide the morphology of the lower extremity arteries for diagnosing PAD [3]. However, the diagnostic accuracy of vessel stenosis is still a challenge in some clinical conditions, such as severe calcification [4], inaccurate timing of the contrast bolus due to the long-distance vessels of the lower extremities, asymmetric proximal stenoses or abnormal cardiac function [5].

Dynamic CTA (dyn-CTA) of the lower extremities offers a solution for patients with diagnostic inaccuracy problems involving s-CTA [6]. In recent years, as new examination techniques, continuous bidirectional table movements have enabled dynamic volume coverage in an area up to 45 cm long and achieve whole dynamic imaging of the lower legs [7]. Feasibility studies of dyn-CTA of the vessels the beneath knees have provided promising results [8]. Compared with s-CTA, dyn-CTA shows better performance on arterial contrast enhancement, better diagnostic confidence, and better diagnostic accuracy in detecting vessel stenoses and occlusions in PAD patients [9]. Buls et al. evaluated the mean CT values (HU) of all arteries below the knees and concluded that dyn-CTA showed higher image quality and diagnostic confidence for assessing the occurrence and degree of arterial stenosis [10].

Most existing studies on dyn-CTA require multiphase data review, which is more time-consuming than the steps required for s-CTA and limits the clinical application of dyn-CTA. Maximum intensity projection (MIP) was introduced for clinical use with CTA [11], and it is widely used in vascular imaging of the whole body [12–15]. Time MIP (t-MIP) images reflect the maximum value of each matrix in the dynamic data for all time phases. It was first described by Murayama et al. [16] for detecting early ischemic changes in patients with acute ischemic stroke. T-MIP was shown to have a better signal noise ratio in white and gray matter of the brain than single-phase CTA [16, 17]. To date, there have been no studies on t-MIP processed from dyn-CTA in facilitating the diagnosis of PAD.

In this study, we used t-MIP CTA to provide an intuitive, fast and noninvasive solution for the diagnosis of lower extremity stenosis. The aim of this study was to assess the diagnostic accuracy of t-MIP CTA postprocessed from dyn-CTA versus that of s-CTA.

## Methods

### Patients

The Institutional Review Board of Peking Union Medical College Hospital approved this study (HS-934). From November 2015 to March 2016, 35 patients with known PAD were included. One patient was excluded due to severe calcified plaques and motion artifacts. Thirty-four patients (average age = 65.4 ± 11.6 years old; 11 females, 23 males; average body mass index = 23.2 ± 3.0) with 68 lower extremities and 204 vascular segments were analyzed.

### CTA protocols

A third-generation dual-source dual-energy CT system (Somatom Definition Force, Siemens Healthcare, Forchheim, Germany) was used to perform the scan. The protocol was previously described in a study on lower leg muscle ischemia evaluation by Zhou et al. [18]. The scan consisted of dyn-CTA and s-CTA. First, dyn-CTA was performed on the lower legs with a 45 cm scan range using shuttle mode. For all scans, automatic tube-current modulation (CARE Dose4D, Siemens Healthcare, Germany) was used. The scan parameters were a tube voltage 70 kV, tube current reference 80 mA and collimation 2 × 64 × 0.6 mm. There were 9 phases of the dyn-CTA scan. The first 5 phases were 2.5 s/phase, and the last 4 phases were 5 s/phase. The data acquisition time was 30 s in total. The algorithm called advanced modeled iterative reconstruction (ADMIRE; Siemens Healthcare, Forchheim, Germany) with soft convolution kernel (Bv40) reconstructed images were rendered with a slice thickness of 1.5 mm and an increment of 1 mm for all 9 acquisition phases. Thirty milliliters of contrast media (iopromide 370 mgI/mL) were injected at a flow rate of 4.0 mL/s, and a saline bolus of 50 mL/s followed at the same flow rate.

S-CTA was performed five minutes later. The scan parameters [19] were a tube voltage 70 kV, tube current reference 322 mA, pitch 0.6, and rotation time 0. 25 s and collimation 2 × 64 × 0.6 mm. The rest scan parameters were same with dyn-CTA. Fifty milliliters of contrast agent (iopromide 370 mgI/mL) were administered intravenously at a flow rate of 2.5 mL/s, and 40 mL saline followed at the same flow rate. The s-CTA scan implemented the bolus tracking technique by placing the region of interest (ROI) at the healthy popliteal artery. When the threshold reached 100 HU, the scan started automatically after 6 s. Soft convolution kernel (Bv40) reconstructed images were rendered with a 1.5 mm slice thickness and a 1 mm increment.

### CT radiation dose

To estimate the CTA radiation dose, the volume CT dose index (CTDIvol) and the dose length product (DLP) from the dose report of each patient were documented. Since there were no conversion coefficients k for the effective dose of CTA in the lower extremities of 70 kV, no effective dose was calculated [20].

### Data postprocessing

**Fig. 1:**
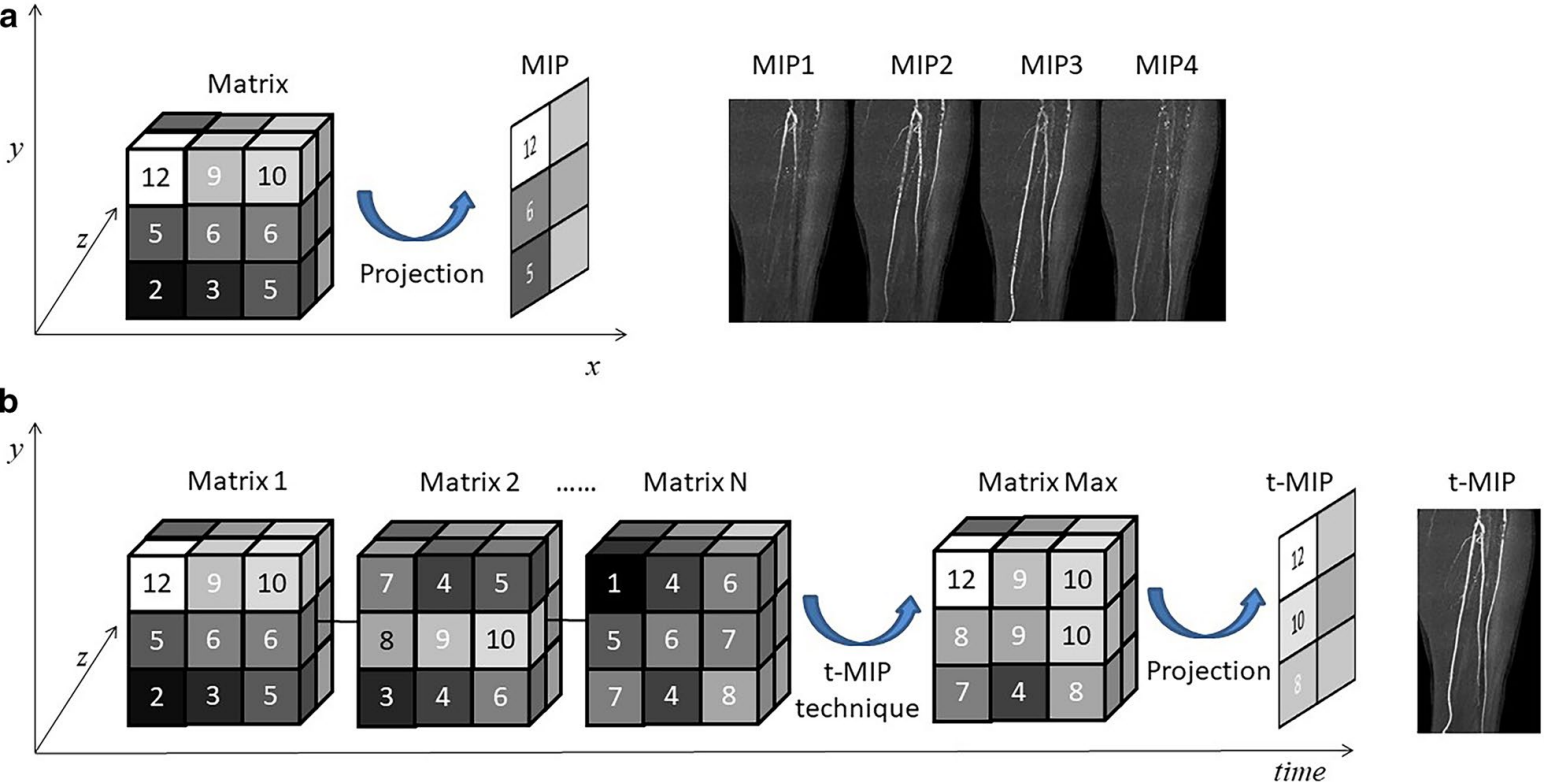
Schematic diagram and an example of dyn-CTA of the lower legs with MIP (**a**) and t-MIP (**b**) postprocessing techniques. MIP preserved the maximum value of one matrix. For t-MIP, the maximum value of each matrix at each time point of dyn-CTA was preserved and merged into one large matrix. dyn-CTA, dynamic computed tomographic angiography; t-MIP, time maximum intensity projection

Dyn-CTA MIP generation was performed using MATLAB R2017a (MathWorks, Natick, MA) with in-house software. DICOM data were loaded into the software. To ensure acquisition of clear vascular images, 1.5 mm thick data were used for analysis, with 453 slices in total. First, the patient bed was removed from the source data. Then, bone was automatically removed from the source data by using a threshold value equal to 5 times the average CT attenuation of the whole image. The maximum value of each matrix was reserved for the vasculature, muscle and fat. All phase images were arranged in line and merged into one large matrix. Finally, for each lower extremity, the axial images and three t-MIP images (Fig. 1)—MIP_0_ (sagittal MIP), MIP_+θ_ (45° lateral MIP), and MIP_−θ_ (−45° lateral MIP)—were automatically generated from the large matrix for diagnosis. The analysis process attempted to avoid interference from human factors. The images were processed by a single medical physicist who was blinded to the patient groupings.

### Data analysis

#### Objective analysis

The CT attenuation of three lower extremity artery segments (anterior tibial artery, posterior tibial artery and fibular artery) was measured by one radiologist (DZ, 8 years of experience) on axial t-MIP CTA images using the best enhancement phase of dyn-CTA and s-CTA. ROIs were placed at the proximal third of the lower legs on the same slice for t-MIP CTA, dyn-CTA and s-CTA.

#### Subjective analysis

For each of the three lower extremity artery segments, stenosis percentage and occlusion length were evaluated in the form of a runoff score. This score ranged from 0 to 9, with a higher score indicating more severe disease. For each of the three lower extremity artery segments, the score was assigned as follows: 0, no-20% stenosis; 1, 21–49% stenosis; 2, 50–99% stenosis, 2.5, < half of the vessel length occluded; and 3, > half of the vessel length occluded. All 3 vessel scores were added together to determine the runoff score for the lower extremities [21].

The scores were provided by two vascular imaging radiologists (DZ and HZ with 3 years of experience). The s-CTA images were evaluated first to ensure memory washout. Four weeks later, the t-MIP CTA images were provided to two vascular imaging radiologists, and they gave scores respectively with the same evaluation criteria as for the s-CTA images.

According to the runoff score of the s-CTA images, 68 lower legs were divided into a normal group (n = 24) with each vessel segment score ≤ 1 and a runoff score ≤ 2 and an abnormal group with vascular stenosis (n = 44).

### Statistical analyses

All statistical analyses were performed using the Statistical Package for Social Sciences, version 19.0 (SPSS Inc., Chicago, IL, USA). Continuous variables were expressed as the mean ± standard deviation (SD). Normality of data distribution was assessed using the Kolmogorov­ Smirnov test. The difference in normally distributed numerical data sets among the three groups was tested by using ANOVA. When the ANOVA results were significant, Tukey’s honest significant distance (HSD) procedure was used for multiple comparisons between the three groups in a pairwise manner. The Kruskal–Wallis rank-sum test was used to assess the differences in nonnormally distributed data among the three groups. When the result of the Kruskal–Wallis test was significant, the Mann–Whitney U test was used for pairwise comparisons. Categorical data were compared with the paired Wilcoxon signed-rank test. A Bland–Altman outlying plot was used to assess the consistency of the runoff scores between t-MIP and s-CTA. Numbers that were within the mean plus or minus 1.96 times the standard deviation were usually not emphasized. A p-value less than 0.05 was considered significant. Cronbach’s alpha (α) was calculated for measuring interobserver agreement among the two radiologists.

## Results

The indication for CTA was limb ischemia (Fontaine stage I, n = 6; Fontaine stage II, n = 19; Fontaine stage III, n = 3; and Fontaine stage IV, n = 6). The mean ± SD of CTDIvol and DLP were, respectively, 9.1 ± 0.0 mGy and 396.9 ± 0.1 mGy × cm for dyn-CTA and 1.6 ± 0.3 mGy and 212.4 ± 41.5 mGy × cm for s-CTA.

**Fig. 2:**
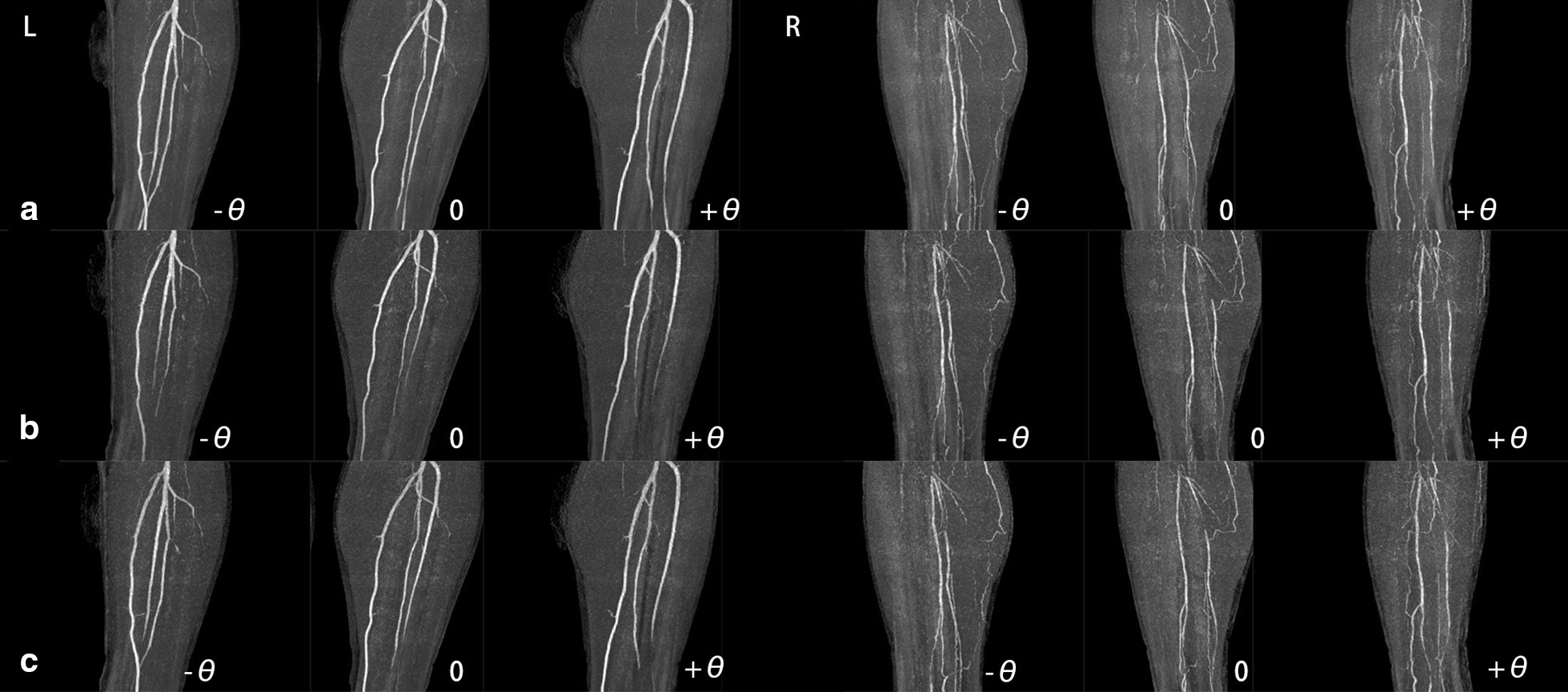
Comparison of t-MIP CTA and different phases of dyn-CTA of the lower extremities for a male patient with ischemia of the right lower extremity and normal left lower extremity. **a** The t-MIP images, and the proximal and distal vessel segments of the arteries of the lower legs show optimal enhancement. **b** and **c** MIP images of the second and third phases of dyn-CTA. The proximal vessel segments are more enhanced in the second phase, while the distal vessel segments are more enhanced in the third phase. 0, sagittal position; + θ, 45 ° lateral position; −θ, −45° lateral position

In clinical practice, it is difficult to acquire satisfactory MIP images from patients with asymmetric vascular stenosis and different peak enhancement times of the lower extremities. Different phases of dyn-CTA are required to achieve peak enhancement of different segments of lower extremity vessels. T-MIP merges all phases of dyn-CTA and generates the optimal enhancement for both proximal and distal vessels (Fig. 2).

### Objective analysis

The CT attenuation of t-MIP CTA of the three vascular segments from 68 lower extremities was higher than that of the best enhancement phase of dyn-CTA and s-CTA (Table 1). For CT attenuation of the anterior tibial artery, there was no significant difference between the three groups (*p* = 0.135). For the posterior tibial artery and fibular artery, there was a significant difference between the CT attenuation of t-MIP CTA and s-CTA (all *p* < 0.05). For the average CT attenuation of the anterior tibial artery, posterior tibial artery and fibular artery, there was a significant difference between t-MIP CTA and s-CTA as well as between dyn-CTA and s-CTA (all *p* < 0.05).

**Table 1:** CT attenuation of the anterior tibial artery, posterior tibial artery and fibular artery for t-MIP and the best enhancement phase of dyn-CTA and s-CTA

CT attenuation (HU)	t-MIP	Dyn-CTA	s-CTA
Anterior tibial artery	375.6 ± 148.0	347.3 ± 120.4	319.5 ± 112.6
Posterior tibial artery	371.1 ± 105.9	344.6 ± 101.9	320.6 ± 97.8
Fibular artery	343.8 ± 94.0	322.3 ± 108.3	287.5 ± 107.4
Average	363.4 ± 117.9	337.6 ± 110.3	308.5 ± 106.1

t-MIP, time maximum intensity projection; dyn-CTA, dynamic computed tomographic angiography; s-CTA, standard computed tomographic angiography

### Subjective analysis

There was good interobserver agreement for the assigned runoff score based on t-MIP images between the two radiologists (α = 0.833).

**Fig. 3:**
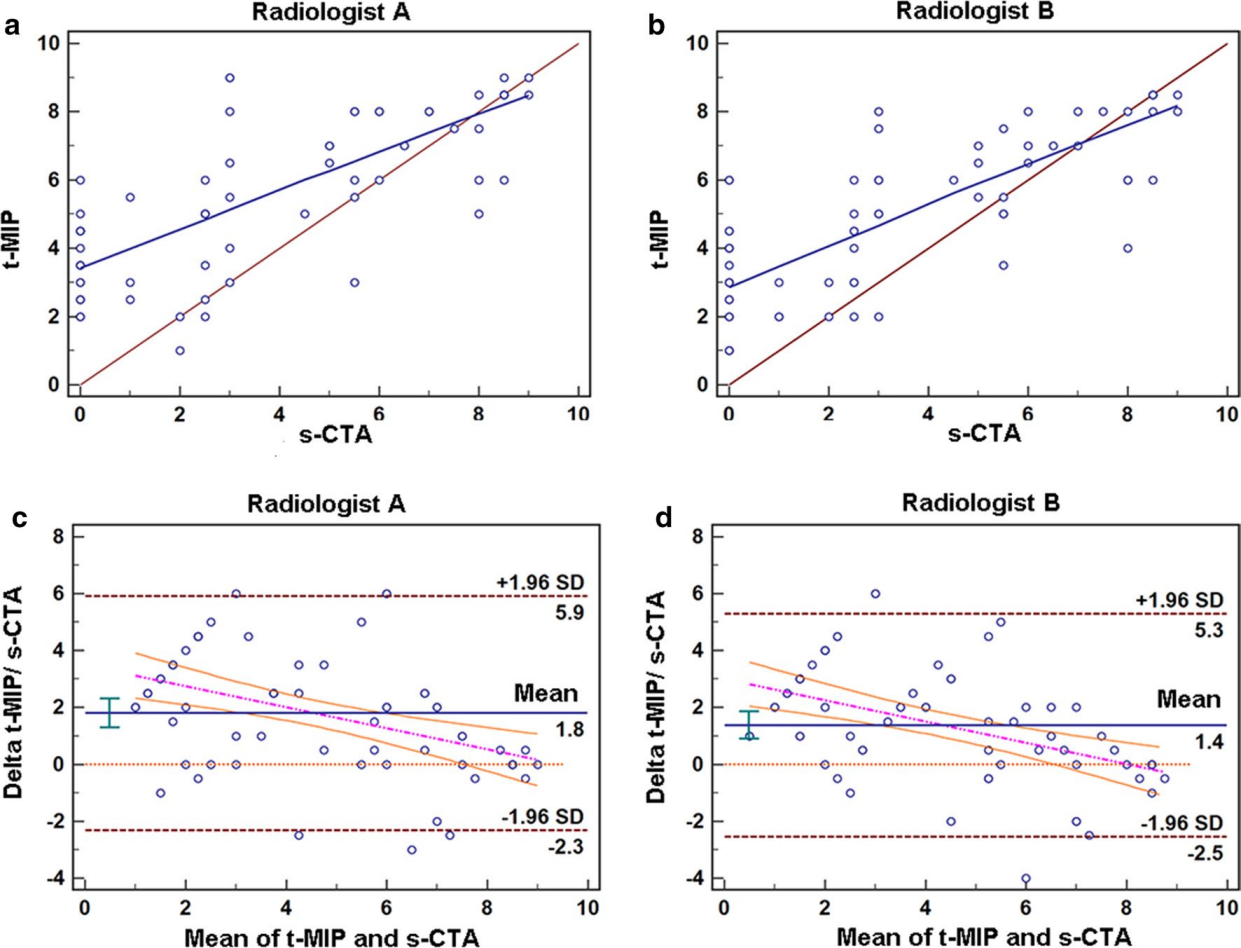
Comparison of runoff scores between t-MIP and s-CTA. **a**, **b** Regression curve, runoff score of t-MIP by radiologist A = 0.54, runoff score of s-CTA = 3.49; r = 0.75; p < 0.001. Runoff score of t-MIP by radiologist B = 0.57, runoff score of s-CTA = 3.01; r = 0.78; p < 0.001. **c**, **d** Bland–Altman plots, horizontal lines indicate the mean difference and 95% limits of agreement (95% LAs)

The runoff scores evaluated with t-MIP and s-CTA were correlated for both radiologist A (r = 0.75, p < 0.001) and radiologist B (r = 0.78, p < 0.001) (Fig. 3). The results of Bland–Altman analysis are summarized in Table 2, showing a mean difference of 1.79 and 95% limits of agreement of − 2.32 to 5.91 for radiologist A and a mean difference of 1.38 and limits of agreement of − 2.55 to 5.30 for radiologist B. The runoff score per leg from s-CTA was 3.7 ± 3.2. Compared with that from s-CTA, the runoff score per leg from t-MIP for radiologists A and B was significantly different (radiologist A, 5.6 ± 2.3, p < 0.001; radiologist B, 5.1 ± 2.3, p < 0.001).

**Table 2:** Comparison between t-MIP and s-CTA runoff scores using Bland–Altman analysis

		Radiologist A	Radiologist B
Correlation	r	0.75	0.78
	p	< 0.001	< 0.001
Regression line	Slope	0.54	0.57
	y_0_	3.49	3.01
Difference (t-MIP-s-CTA)	Mean ± SD	1.79 ± 2.10	1.38 ± 2.00
	95% LA	− 2.32 to 5.91	− 2.55 to 5.30
	SEM	0.47	0.43
	95% CI	1.29 to 2.30	0.89 to 1.86
	Bias	Yes	Yes

t-MIP, time maximum intensity projection; s-CTA, standard computed tomographic angiography; SD, standard deviation; 95% LA, 95% limits of agreement; SEM, standard error of the mean difference; 95% CI; 95% confidence interval

The patient’s runoff score from both s-CTA and t-MIP was higher when he or she was at a more severe clinical stage (Table 3). For radiologist B, the t-MIP runoff score was significantly higher (*p* = 0.034) for patients with Fontaine stage III + IV than for those with Fontaine stage I + II. For radiologist A, there was no significant difference in either the s-CTA or t-MIP runoff scores between patients with Fontaine stage I + II and Fontaine stage III + IV.

**Table 3:** Runoff scores and clinical stages of t-MIP and s-CTA

Runoff score	Fontaine stage I + II	Fontaine stage III + IV
t-MIP radiologist A	5.7 ± 2.0	7.1 ± 2.3
t-MIP radiologist B	5.0 ± 2.2	6.6 ± 2.3
s-CTA	3.6 ± 3.2	5.9 ± 3.5

t-MIP, time maximum intensity projection; s-CTA, standard computed tomographic angiography

## Discussion

The evaluation of lower extremity vascular stenosis using t-MIP is feasible based on the results of this study. There was a strong correlation between the t-MIP and s-CTA runoff scores. T-MIP images automatically retrieved and merged the maximal CT attenuation of the lower leg arteries from the multiple phases of CTA data and provided an intuitive and clear view for diagnosis.

Studies on dyn-CTA have shown that compared with s-CTA, it can improve arterial enhancement and diagnostic confidence [9, 10], which may compensate for the unsatisfactory arterial enhancement of lower extremity runoff CTA. In addition to the advantage of tracking the best time of the bolus, the large image data of multiphase dyn-CTA consume more time than those of s-CTA and restrict its clinical application.

T-MIP is a technique derived from head CT perfusion that reflects the maximum value on all projection planes at all time points from CT perfusion [16]. A previous study showed that t-MIP-colored images had better discriminative value (area under curve, 0.811) for the detection of early ischemic changes than CT perfusion cerebral blood volume images, t-MIP gray images and noncontrast CT (NCCT) [16]. Another study conducted by Cao et al. revealed that t-MIP images showed higher vascular attenuation than s-CTA images and were better able to predict acute ischemic stroke [17].

According to our knowledge, there have been no previous studies using t-MIP with lower extremity dyn-CTA, and there is no commercial software that can postprocess lower extremity dyn-CTA images into t-MIP images. Our study is the first to apply t-MIP in PAD diagnosis. In this study, the CT attenuation of the anterior tibial artery, posterior tibial artery and fibular artery at the proximal third of the lower legs in the t-MIP images was 21.5 to 28.3 HU higher than that in the best enhancement phase images of dyn-CTA and was 50.5 to 56.1 HU higher than that in s-CTA images. It is reasonable that t-MIP achieved the highest vascular attenuation from the all-phase dyn-CTA images, and a previous study found that t-MIP [17] and dyn-CTA [10] showed higher vascular attenuation than s-CTA. In our study, t-MIP was correlated with s-CTA, which is consistent with the results of a previous study and a follow-up study [16] showing that t-MIP images had a strong positive correlation with NCCT images. Although we used t-MIP to postprocess the dyn-CTA data, the higher diagnostic performance of dyn-CTA was preserved in the t-MIP images. Sommer et al. [9] reported that compared to s-CTA, dyn-CTA had higher sensitivity and specificity in detecting stenosis and occlusion. In this study, the average runoff score of t-MIP was higher than that of s-CTA, which indicates a severe level of stenosis or occlusion.

The application of dyn-CTA of the lower extremities was also restricted by the long scan range and high radiation dose. Newer CT scanners provide dynamic CTA with scan ranges up to 60 cm in length in shuttle mode, which is useful for lower leg dyn-CTA. In addition to a longer scan range, the tube voltage of 70 kV in runoff CTA helped reduce the radiation dose in previous studies [22, 23], as did the 45 mL of contrast medium, which is less than half the amount used in routine runoff CTA [22]. In this study, a low tube voltage of 70 kV for dyn-CTA resulted in a DLP of 396.9 ± 0.1 mGy × cm. The contrast medium volume was relatively large for the lower extremities. To prevent renal function impairment [24], the contrast medium was reduced to 30 mL for dyn-CTA and 50 mL for s-CTA. The DLP and the 80 mL contrast volume together make combining dyn-CTA and runoff CTA more applicable in clinical practice.

There were several limitations to this study. First, the population of patients was small, which made subgrouping based on different clinical stages impossible. Further studies should involve more patients, especially patients with Fontaine stage III and IV, for whom the contrast bolus time may be more likely abnormal. Second, the runoff score of t-MIP was only compared with that of s-CTA, and a gold-standard analysis was lacking. This was partly because of the small patient population, particularly the small number of patients with a severe clinical stage. Third, the postprocessed images of t-MIP not only retrieved the highest vascular CT attenuation but also selected muscle CT attenuation. Although the enhancement of muscle was mild, it may still lower the contrast-to-noise ratio of t-MIP.

## Conclusions

In conclusion, there is potential clinical application of t-MIP in assisting with the diagnosis of lower leg vascular stenosis in dyn-CTA, with more reliable diagnostic accuracy than s-CTA and more convenient immediacy than dyn-CTA. Therefore, t-MIP is a powerful noninvasive and quick diagnostic method that can be used in the treatment plans of PAD patients.

## Data Availability

The datasets used and/or analyzed during the current study available from the corresponding author on reasonable request.

## Abbreviations

PAD: Peripheral arterial disease
CTA: Computed tomographic angiography
S-CTA: Standard CTA
Dyn-CTA: Dynamic CTA
MIP: Maximum intensity projection
T-MIP: Time MIP
CTDIvol: Volume CT dose index
DLP: Dose length product
SD: Standard deviation
HSD: Honest significant distance
NCCT: Noncontrast CT

## References

[1] Patel AY, Gurm HS: Medical management of lower extremity peripheral artery disease. In: Practical approach to peripheral arterial chronic total occlusions. edn.: Springer; 2017: 1–8.

[2] Dua A, Lee CJ (2016). Epidemiology of peripheral arterial disease and critical limb ischemia. Tech Vasc Interventional Radiol.

[3] Preuss A, Schaafs LA, Werncke T, Steffen IG, Hamm B, Elgeti T (2016). Run-off computed tomography angiography (CTA) for discriminating the underlying causes of intermittent claudication. PLoS ONE.

[4] Pollak AW, Norton PT, Kramer CM (2012). Multimodality imaging of lower extremity peripheral arterial disease: current role and future directions. Circ Cardiovasc Imaging.

[5] Keeling AN, Farrelly C, Carr JC, Yaghmai V (2011). Technical considerations for lower limb multidetector computed tomographic angiography. Vasc Med.

[6] Werncke T, Ringe KI, von Falck C, Kruschewski M, Wacker F, Meyer BC (2015). Diagnostic confidence of run-off CT-angiography as the primary diagnostic imaging modality in patients presenting with acute or Chr. PLoS ONE.

[7] Sommer WH, Helck A, Bamberg F, Albrecht E, Becker CR, Weidenhagen R, Kramer H, Reiser MF, Nikolaou K (2010). Diagnostic value of time-resolved CT angiography for the lower leg. Eur Radiol.

[8] Kortman HGJ, Smit EJ, Oei MTH, Manniesing R, Prokop M, Meijer FJA (2015). 4D-CTA in neurovascular disease: a review. Am J Neuroradiol.

[9] Sommer WH, Bamberg F, Johnson TR, Weidenhagen R, Notohamiprodjo M, Schwarz F, Reiser MF, Nikolaou K (2012). Diagnostic accuracy of dynamic computed tomographic angiographic of the lower leg in patients with critical limb ischemia. Invest Radiol.

[10] Buls N, de Brucker Y, Aerden D, Devos H, van Gompe G, Boonen PT, Nieboer K, Leiner T, de Mey J (2019). Improving the diagnosis of peripheral arterial disease in below-the-knee arteries by adding time-resolved CT scan series to conventional run-off CT angiography. First experience with a 256-slice CT scanner. Eur J Radiol.

[11] Napel S, Marks MP, Rubin GD, Dake MD, McDonnell CH, Song SM, Enzmann DR, Jeffrey RB (1992). CT angiography with spiral CT and maximum intensity projection. Radiology.

[12] Galanski M, Prokop M, Chavan A, Schaefer CM, Jandeleit K, Nischelsky JE (1993). Renal arterial stenoses: spiral CT angiography. Radiology.

[13] Iglesias J, Pena C (2014). Computed tomography angiography and magnetic resonance angiography imaging in critical limb ischemia: an overview. Tech Vasc Interven Radiol.

[14] Jeong YJ, Lee KS, Yoon YC, Kim TS, Chung MJ, Kim S (2004). Evaluation of small pulmonary arteries by 16-slice multidetector computed tomography: optimum slab thickness in condensing transaxial images converted into maximum intensity projection images. J Comput Assist Tomogr.

[15] Randoux B, Marro B, Koskas F, Duyme M, Sahel M, Zouaoui A, Marsault C (2001). Carotid artery stenosis: prospective comparison of CT, three-dimensional gadolinium-enhanced MR, and conventional angiography. Radiology.

[16] Murayama K, Suzuki S, Matsukiyo R, Takenaka A, Hayakawa M, Tsutsumi T, Fujii K, Katada K, Toyama H (2018). Preliminary study of time maximum intensity projection computed tomography imaging for the detection of early ischemic change in patient with acute ischemic stroke. Medicine.

[17] Cao R, Jiang Y, Lu J, Wu G, Zhang L, Chen J. Evaluation of intracranial vascular status in patients with acute ischemic stroke by time maximum intensity projection CT . Acad Radiol. 2019;27:696. 10.1016/j.acra.2019.06.013 31324580

[18] Zhou X, Zhang D, Zhang H, Lin Z, Fan X, Jin Z (2020). Quantitative analysis of lower leg muscle enhancement measured from dynamic computed tomographic angiography for diagnosis of peripheral arterial occlusive disease. J Comput Assist Tomogr.

[19] Qi L, Meinel FG, Zhou CS, Zhao YE, Schoepf UJ, Zhang LJ, Lu GM (2014). Image quality and radiation dose of lower extremity CT angiography using 70 kVp, high pitch acquisition and sinogram-affirmed iterative reconstruction. PLoS ONE.

[20] Saltybaeva N, Jafari ME, Hupfer M, Kalender WA (2014). Estimates of effective dose for CT scans of the lower extremities. Radiology.

[21] Stoner MC, Calligaro KD, Chaer RA, Dietzek AM, Farber A, Guzman RJ, Hamdan AD, Landry GJ, Yamaguchi DJ (2016). Society for Vascular S: **Reporting standards of the Society for Vascular Surgery for endovascular treatment of chronic lower extremity peripheral artery disease**. J Vasc Surg.

[22] Horehledova B, Mihl C, Milanese G, Brans R, Eijsvoogel NG, Hendriks BMF, Wildberger JE, Das M (2018). CT Angiography in the lower extremity peripheral artery disease feasibility of an ultra-low volume contrast media protocol. Cardiovasc Intervent Radiol.

[23] Qi L, Zhao Y, Zhou CS, Spearman JV, Renker M, Schoepf UJ, Zhang LJ, Lu GM (2015). Image quality and radiation dose of lower extremity CT angiography at 70 kVp on an integrated circuit detector dual-source computed tomography. Acta Radiol.

[24] Andreucci M, Faga T, Pisani A, Sabbatini M, Michael A (2014). Acute kidney injury by radiographic contrast media: pathogenesis and prevention. Biomed Res Int.

